# Factors associated with older adults’ cognitive decline 6 months after gamma-variant SARS-CoV-2 infection

**DOI:** 10.3389/fneur.2024.1334161

**Published:** 2024-02-15

**Authors:** Vanessa Giffoni M. N. P. Peixoto, Lucas Alves Facci, Thiago C. S. Barbalho, Raíssa Nascimento Souza, Alice Mendes Duarte, Marina Bruxel dos Santos, Katie Moraes Almondes

**Affiliations:** ^1^Post-graduation Program in Psychobiology, Universidade Federal do Rio Grande do Norte, Natal, Brazil; ^2^Department of Clinical Medicine, Universidade Federal do Rio Grande do Norte, Natal, Brazil; ^3^Universidade Federal do Rio Grande do Norte, Natal, Brazil; ^4^Department of Psychology, Universidade Federal do Rio Grande do Norte, Natal, Brazil

**Keywords:** cognitive decline, cognition, older adults, COVID-19, SARS-CoV-2, post-COVID syndrome, long-term

## Abstract

**Background:**

Cognitive deficits are commonly reported after COVID-19 recovery, but little is known in the older population. This study aims to investigate possible cognitive damage in older adults 6 months after contracting COVID-19, as well as individual risk factors.

**Methods:**

This cross-sectional study involved 70 participants aged 60–78 with COVID-19 6 months prior and 153 healthy controls. Montreal Cognitive Assessment—Basic (MoCA-B) screened for cognitive impairment; Geriatric Depression Scale and Geriatric Anxiety Inventory screened for depression and anxiety. Data were collected on demographics and self-reports of comorbid conditions.

**Results:**

The mean age of participants was 66.97 ± 4.64 years. A higher proportion of individuals in the COVID group complained about cognitive deficits (χ^2^ = 3.574; *p* = 0.029) and presented with deficient MoCA-B scores (χ^2^ = 6.098, *p* = 0.014) compared to controls. After controlling for multiple variables, all the following factors resulted in greater odds of a deficient MoCA-B: COVID-19 6-months prior (OR, 2.44; *p* = 0.018), age (OR, 1.15; *p* < 0.001), lower income (OR, 0.36; *p* = 0.070), and overweight (OR, 2.83; *p* = 0.013). Further analysis pointed to individual characteristics in COVID-19-affected patients that could explain the severity of the cognitive decline: age (*p* = 0.015), lower income (*p* < 0.001), anxiety (*p* = 0.049), ageusia (*p* = 0.054), overweight (*p* < 0.001), and absence of cognitively stimulating activities (*p* = 0.062).

**Conclusion:**

Our study highlights a profile of cognitive risk aggravation over aging after COVID-19 infection, which is likely mitigated by wealth but worsened in the presence of overweight. Ageusia at the time of acute COVID-19, anxiety, being overweight, and absence of routine intellectual activities are risk factors for more prominent cognitive decline among those infected by COVID-19.

## 1 Introduction

The disease caused by the SARS-CoV-2 virus has been the main public health concern for more than 3 years. Extensive literature has been published on the clinical aspects of the acute disease (1), and although the end of the COVID-19 public emergency was declared on May 5, 2023 (2), uncertainties about the long-term nature of sequelae that survivors might face still exist. As so, many longitudinal studies have been looking into post-covid symptoms, their predictors, and whether they might impact patients transiently or in a definitive way (3).

COVID-19 may affect the central nervous system in distinct ways, which may combine in some individuals. Neuroinflammation may be triggered by the acute infection and perpetuated chronically, resulting in increased blood–brain-barrier (BBB) permeability, persistent elevated inflammatory cytokines and chemokines, and hyperactivation of microglia and other brain cells (4). Severe pulmonary or vascular disease can lead to hypoxic damage, which is often worsened by multi-organ dysfunction (5). Finally, there is very limited but available evidence that the virus can directly invade the brain (6). Consequently, neurological and psychiatric problems are commonly reported long after COVID-19 recovery, even in those experiencing mild disease (7). In a recent systematic review and meta-analysis, 50.1% of survivors (95% CI 45.4–54.8) had at least one physical or mental symptom up to 12 months after the acute infection, in which the combined prevalence of cognitive deficits was 19.7% (8.8–33.4), 17.5% for memory impairment (8.6–29.6), and 12.6% for concentration impairment (5.9–21.3) (8). Intriguingly, data showed that memory complaints were twice greater in younger individuals compared to those aged 60 and over and that asymptomatic and milder cases tended to present more persistent neuropsychiatric symptoms than those who were hospitalized and experienced moderate and severe disease.

Evidence from post-COVID-19 electronic records of more than 242,000 adults over 65 years pointed to an increased cumulative risk of cognitive deficits and dementia when compared to cohorts affected by other respiratory infections, with new diagnoses still occurring up to 2 years after the index event (9). Although this study relied solely on electronic records of new diagnoses and did not involve any participant interviews, concerns have arisen facing the current scenario of 55 million people living with dementia (10) and the expected burden of 10 million new cases annually, especially in low and middle-income countries, in the pre-COVID-19 era (11). If dementia risk is persistently higher [HR 1.33 (1.26–1.41)] among older individuals affected by SARS-CoV-2 (9), then clinicians and public health agencies must be prepared to deal with a wave of cognitively impaired individuals that may arise following COVID-19 pandemic.

It is imperative to mention that a few studies are focused exclusively on the older age group, which is highly susceptible to cognitive disorders. Furthermore, there is insufficient literature regarding post-COVID symptoms related to the gamma variant of SARS-CoV-2. We hypothesize that the virus leads to cognitive impairment, and advanced age might be a predictor of this outcome. Therefore, this study aims to investigate (1) if older adults experience cognitive decline 6 months after contracting COVID-19 and (2) whether individual underlying conditions may influence this outcome.

## 2 Methods

### 2.1 Study design and population

This is a cross-sectional analysis of a longitudinal cohort study with a total follow-up of 2 years, comprising two groups of older adults, either previously infected by SARS-CoV-2 or not (controls). Based on a ratio of 4/8 between unexposed and exposed, a significance level of 95%, a power of 80%, and accounting for a 20% loss, the minimum required sample size was estimated to be 77 participants. Recruitment took place in March 2021, during the second wave of the pandemic, in which the Gamma variant was circulating. Eligible participants were those aged 60 to 80 with a formal education of over 4 years. Individuals with previous cognitive decline, uncontrolled psychiatric conditions, recent stroke, heart attack, or cardiac arrest were excluded. The general inclusion and exclusion criteria were applied to both cohorts. COVID (+) participants were recruited from a private laboratory in Natal, Brazil, among all 527 patients aged 60 to 80 diagnosed with SARS-CoV-2 infection by RT-PCR technique in March 2021. Healthy controls, which means, those who had never been infected by SARS-CoV-2, were engaged through social media advertisements in the same month and city; their serologic status was tested through the ELISA technique to rule out previously unknown disease. None of the participants had received any COVID-19 vaccine yet. Of 527 COVID candidates, 153 volunteered to join the study, but only 70 composed the COVID group after general inclusion and exclusion criteria were applied. Of the 214 volunteers who answered the social media announcement for the CONTROL group, 153 were recruited through the same criteria. Due to the longitudinal nature of the study and the anticipated proportion of controls to contract COVID-19 during the follow-up, a final of 70 COVID and 153 control participants comprised the initial sample. The data presented in this paper was collected between May and September 2021, concerning the first of three assessments of the prospective follow-up. In the COVID group, 6 months elapsed from the diagnosis to the assessment. A certified geriatrician, neuropsychologist, and trained research assistants conducted the assessments at the Institute of Tropical Medicine, Federal University of Rio Grande do Norte (UFRN).

### 2.2 Instruments

A structured questionnaire was used to record data on demographics (age, gender, marital status, education, family income, occupational status, routine activities), subjective memory complaints, self-report of comorbid conditions, and clinical characteristics of acute and post-COVID-19 symptoms (for the COVID group). Regarding income, we collected respondents’ information on individual family amounts, which were summed up to estimate the aggregated total family income. The income ranges were divided according to multiples of the Brazilian minimum monthly wage (12). Depression was screened by the Geriatric Depression Scale (GDS-15), ranging from 0 to 15, with scores >5 indicating depression. Anxiety was suggested by scores higher than 10 on the Geriatric Anxiety Inventory (GAI) (13). Pfeffer’s Functional Activities Questionnaire (FAQ) score > 6 and the Telephone Interview for Cognitive Screening (TICS-m) < 21 were used to rule out participants with previous dementia. TICS-m is a 13-item assessment (maximum score is 39) of four domains: orientation; memory (registration, recent memory and delayed recall); attention/calculation; language (semantic memory, comprehension and repetition) (14, 15). Global cognitive function was tested through the Brazilian version of the Montreal Cognitive Assessment – Basic version (MoCA-B) – adopted in this study to better evaluate individuals with lower educational levels. MoCA-B assesses eight domains: attention and concentration; executive functions; memory; language; visuoconstructional skills; conceptual thinking; calculations, and orientation (16). The maximum score is 30 points, and to represent the most relevant metric of MoCA-B for clinical practice, the performances were binary classified as normal, whenever the score from the test was at least 25, or deficient, otherwise.

### 2.3 Statistics

Descriptive and inferential analyses were completed for demographic and clinical data, GDS-15, GAI, TICS-m, and MoCA-B. According to the Shapiro–Wilk test, normally distributed continuous variables were analyzed by Student’s *t*-test or Mann–Whitney test for non-normally distributed variables. Pearson’s chi-square or Fisher’s tests were used for group comparisons on nominal variables. The purpose of testing (and estimating) the effect of COVID-19 on cognitive function as addressed by MoCA-B was pursued by fitting logistic regression models on the scopus of parametric statistical modeling. Once such an objective had been achieved, statistical modeling was also used to probe for COVID-19 manifestations that could explain (or predict) the severity of cognitive symptoms, this time grounded on the class of cumulative link models (CLM), being the expected cumulative probability of a given outcome linked to a linear combination of the predictors by the logit function. In both circumstances, the statistical modeling was guided by previous extensive exploratory analysis to unveil tentative patterns and relationships to be further tested in a formal inferential context. In addition, these qualitative preliminary steps allowed us to outline the correlation structure among putative predictors so that multicollinearity could be avoided. On the basis of the exploratory information, plausible models were fitted to data, then validated and selected according to the goodness of fit and the suitability to address the scientific hypothesis supporting this study. In this regard, model validation proceeded according to the tools available for each model class. Thus, for logistic models, validation was grounded chiefly on leverage and influence measures as well as the analysis of residuals’ envelope. When it came to CLM, validation was supported by metrics such as Akaike Information Criteria (AIC) and conditional Hessian. CLM was fitted under the assumptions of flexible thresholds and constant odds. The latter implies that the odds for a greater score are the same regardless of the level of the MoCA-B scale. Finally, the statistical significance of any association was conditioned to the significance level *α* = 0.05, and all the codes, analyses, and graphical elements were elaborated in RStudio software.

### 2.4 Ethics statement

The study was conducted according to ethical guidelines after approval by the local Research Ethics Committee of UFRN, under process number 44011221.8.0000.5537. Written informed consent was collected from all participants before conducting the study. All information was kept confidential.

## 3 Results

The study involved 223 participants (70 previously exposed to SARS-CoV-2—6 months from the diagnosis to the assessment—"COVID group”; and 153 healthy peers “CONTROL group”). The mean age of participants was 66.97 ± 4,64 years (age range from 60 to 78), and approximately 70% were female. Other sociodemographic characteristics of the sample are summarized in Table 1. The features of acute COVID-19 and persistent symptoms at the time of assessment, as well as the leading comorbidities and medications in use for both groups are listed in Supplementary material.

**Table 1:** Comparison of sociodemographic and other characteristics between COVID and CONTROL groups during the second wave of COVID-19.

Variables	COVID (*n* = 70)	CONTROL (*n* = 153)	Statistics	*p*-value
Age—M ± DP	67.11 ± 4.95	66.90 ± 4.51	0.958^1^	0.813
60–69—n (%)	46 (65.7)	105 (68.6)		
70–78—n (%)	24 (34.3)	48 (31.4)	0.186^2^	0.666
Gender—n (%)			0.692^2^	0.405
Female	46 (65.7)	109 (71.2)		
Male	24 (34.3)	44 (28.8)		
Marital status—n (%)				
Single	01 (1.5)	14 (9.2)	5.894^3^	0.052
Married	50 (71.4)	89 (58.2)		
Divorced	8 (11.4)	27 (17.6)		
Widower	11 (15.7)	23 (15)		
Schooling—n (%)				
4–8 years	2 (2.9)	5 (3.3)	7.578^3^	**0.000**
9 years	6 (8.6)	6 (3.9)		
12 years	25 (35.7)	33 (21.6)		
Higher level	25 (35.7)	68 (44.4)		
Postgraduate	12 (17.1)	41 (26.8)		
Income—n (%)*			9.815^3^	**0.042**
BRL 1,000–1,600	3 (4.3)	3 (2)		
BRL 1,600–3,000	5 (7.1)	19 (12.4)		
BRL 3,000–5,000	14 (20)	23 (15)		
BRL 5,000–10,000	28 (40)	37 (24.2)		
BRL 10,000–15,000	9 (12.9)	32 (20.9)		
BRL 15,000–23,000	8 (11.4)	28 (18.3)		
Over BRL 23,000	3 (4.3)	11 (7.2)		
Occupation—n (%)				
Retired	49 (70)	107 (69.9)		
Working	21 (30)	38 (24.8)	0.716^3^	0.397
Unemployed	0	8 (5.2)		
Routine activities				
Exercising	36 (51.4)	95 (62.1)		
Traveling	50 (71.4)	120 (78.4)		
Handcrafting/gardening	46 (65.7)	97 (63.4)		
Daily Reading	35 (50)	104 (68)		
Cinema/theater	33 (47.1)	99 (64.7)		
Volunteering work	20 (28.6)	53 (34.6)		
Religious practice	18 (25.7)	51 (33.3)		
Crosswords/sudoku	24 (34.3)	71 (46.4)		
Social engagement	34 (48.6)	70 (51,6)		
Playing cards/board games	11 (15.7)	32 (20.9)		
Cognitive training/language/ Technology classes	8 (11.4)	33 (21.6)		
Playing instruments	1 (14.3)	18 (11.8)		
Subjective cognitive complaints—n (%)	40 (57.1)	65 (42.5)	3.574^2^	**0.029**

Values expressed as mean (M) ± standard deviation (SD) or number of participants (n)/frequencies (%). ^1^Mann–Whitney (U) test for comparison between the medians of the groups; ^2^Chi-square test (χ^2^). ^3^Fisher test for differences between groups. Significant results (*p* < 0.05) in bold. COVID, individuals with previous COVID-19; BRL, (Brazilian currency). *Currency 1USD ~ 5BRL.

The COVID group presented lower scores on TICS-m (*U* = 4806.5; *p* = 0.001) and MoCA-B (*U* = 6018.5; *p* = 0.134) compared to controls. Considering a cut-off point 25 for cognitive decline on MoCA-B, a higher proportion of impaired individuals were found in the COVID group (χ^2^ = 6.098, *p* = 0.014; Table 2).

**Table 2:** Comparison of screening test results for cognitive dysfunction, depression, and anxiety (TICS-m/MoCA-B/GDS-15/GAI) between the COVID and control groups (absolute values and respective classification according to cut-off points, when available).

	COVID (*n* = 70)	CONTROL (*n* = 153)	Statistics	*p* value
TICS-m—M ± SD	24.90 ± 3.79	27.23 ± 4,37	4806.5^1^	**0.001**
MoCA-B—M ± SD	25.56 ± 2.71	26.19 ± 2.39	6018.5^1^	0.134
Deficient—n (%)	25 (35.7)	31 (20.3)	6.098^2^	**0.014**
GDS-15—M ± SD	1.97 ± 2.25	2.03 ± 2.1	5,561^1^	0.638
Altered—n (%)	06 (8.6)	12 (7.8)	0.034^2^	0.853
GAI—M ± SD	4.06 ± 4.49	3.59 ± 4.19	5,149^1^	0.640
Altered—n (%)	12 (17.1)	17 (11.1)	1.544^2^	0.214

Values expressed as mean (M) ± standard deviation (SD) or number of participants (n)/frequencies (%). ^1^Mann–Whitney (U) test for comparison between medians. ^2^Chi-square (χ2) test for differences between groups. Significant results (*p* < 0.05) in bold. COVID individuals with previous COVID-19; TICS-m, telephone interview for cognitive status assessment—modified; MoCA-B, Montreal cognitive assessment—basic; GDS-15, geriatric depression scale—15-item version; GAI, geriatric anxiety inventory.

**Figure 1:**
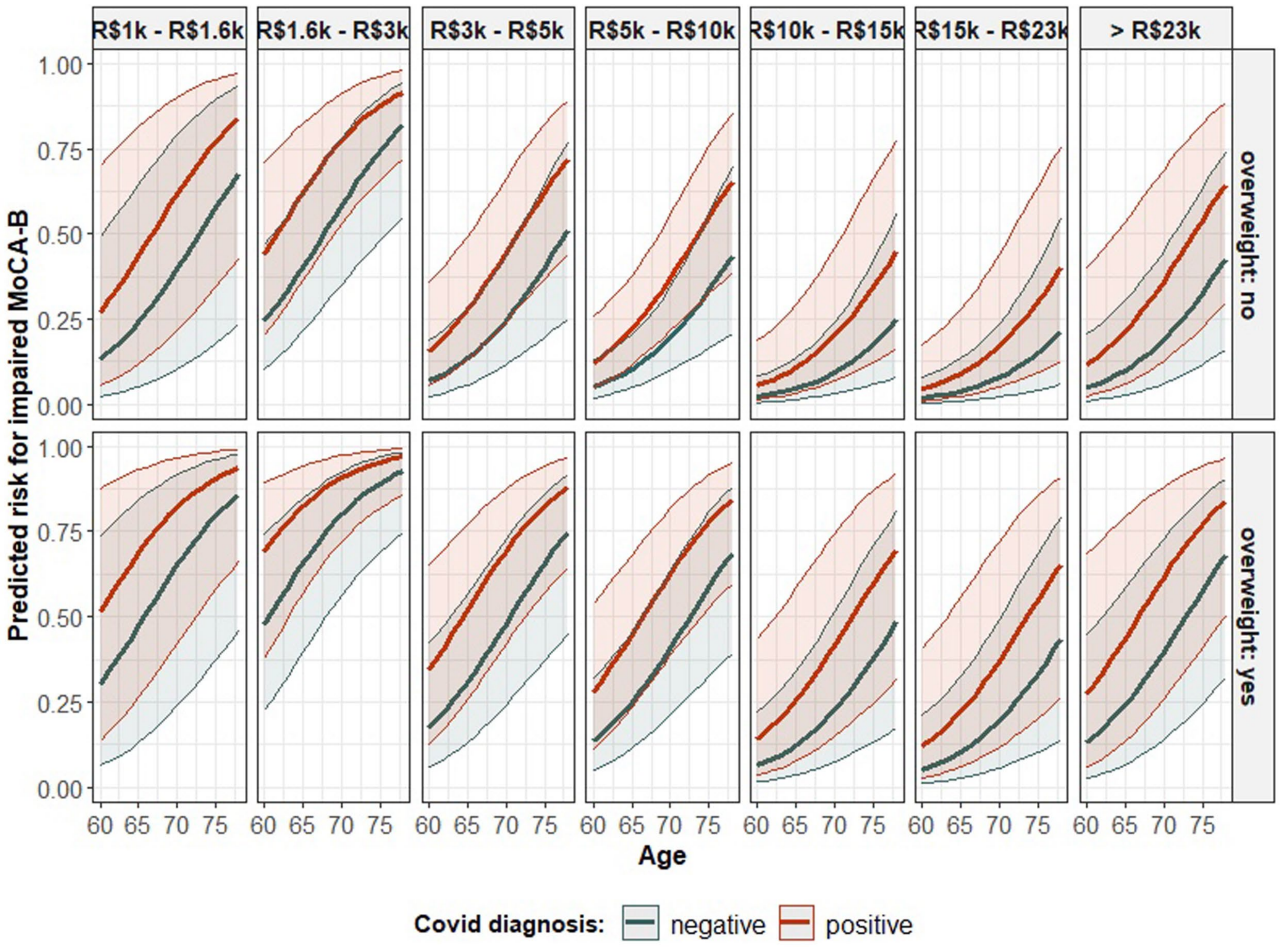
The predicted probability of impaired MoCA-B according to the model in Table 3 - association of age, monthly income, overweight condition, and COVID-19 diagnosis with the performance on such a cognitive test. The statistical parametric model fitted to data to account for the probability of an impaired outcome in the MoCA-B test according to the diagnosis of COVID-19, participant age, income, and overweight status. The class of the model is specified in the table’s head. The estimated coefficients fitting an instance of such a model class are introduced in the “Estimate” column. Each coefficient represents the association between the corresponding predictor (as informed in the row’s label) and the result in the MoCA-B and is followed by the respective statistics from the Wald test for the evaluation of its statistical significance.

The risk for an impaired MoCA-B was found to rely on the diagnosis of COVID-19, as well as on the participant’s age, his/her income, and respective body weight. In this regard, the odds for an impaired outcome were estimated to increase by 15.76% [odds ratio (OR) = 1.157] for every unit increment in the participant age, while all other factors were kept constant. Such an association was based on evidence robust enough to assure its statistical significance (Table 3) (Wald test: estimate = 0.146, std. error (SE) = 0.040, *Z* = 3.606, *p* < 0.001), and it is naturally limited to the age group addressed here (60–78 years old). In addition, while controlling for the other factors, participants’ income was also likely to influence the performance on MoCA-B test, although the evidence provided by the sample to support such an association was insufficient to overcome the significance level, hence remaining as a statistical trend (Table 3) (Wald test: *p* = 0.070 and *p* = 0.054, respectively for the contrasts between R$10 k–R$15 k and R$15 k–R$23 k, both related to R$1 k–R$1.6 k). Even though, as compared to the lowest income range, the odds for an impaired MoCA-B were estimated to reduce by 63.24% (OR = 0.367) and 84.32% (OR = 0.156), respectively, among participants whose reported income was in the range of R$10 k–R$15 k and R$15 k–R$23 k. Moreover, overweight participants were estimated to have 183.44% superior odds (OR = 2.834) for an impaired MoCA-B outcome as compared to normal-weight peers, an association strength estimative controlled by keeping the other factors constant and endorsed by its statistical significance (Table 3) (Wald test: estimate = 1.041, SE = 0.420, *Z* = 2.476, *p* = 0.013). Finally, the risk for impaired MoCA-B was found to be significantly affected by the diagnosis of COVID-19 regardless of the participant’s age, income, and overweight status (Table 3) (Wald test: estimate = 0.895, SE = 0.380, *Z* = 2.354, *p* = 0.018). On this subject, the odds for impaired MoCA-B were estimated to increase by 144.83% (OR = 2.448) once the patient is diagnosed with COVID-19 while controlling for age, income, and overweight condition. Figure 1 depicts these models graphically. The probability of an impaired MoCA-B outcome is estimated to increase as the participant grows older, although the height of the curve seems to depend on income. Except for the highest earnings category (whose incidence was smaller), a trend for lower risk as the income increases seems to take shape. The profile of risk aggravation over aging, which is likely mitigated by wealth, shifts upward as a whole when the participant is overweight. Considering the interaction of social and biological characteristics influencing the result of the MoCA-B test, the association between COVID-19 and cognitive impairment is represented by the vertical distance separating the colored bold curves. In this context, the COVID-19 association with MoCA-B is represented while excluding the bias from age, income, and overweight condition.

**Table 3:** Logistic regression for MoCA-B (Wald test).

	Estimate	Std. Error	*Z*	*p*
(Intercept)	−10.6642	2.8929	−3.6864	2e-04
Covid: positive × negative	0.8954	0.3802	2.3549	0.0185
Age	0.1463	0.0406	3.6068	3e-04
Income: 1.6 k–3 k × 1 k–1.6 k	0.7542	0.969	0.7783	0.4364
Income: 3 k–5 k × 1 k–1.6 k	−0.7044	0.9458	−0.7447	0.4564
Income: 5 k–10 k × 1 k–1.6 k	−1.0008	0.9091	−1.1009	0.2709
Income: 10 k–15 k × 1 k–1.6 k	−1.8527	1.0232	−1.8107	0.0702
Income: 15 k–23 k × 1 k–1.6 k	−2.0509	1.0657	−1.9244	0.0543
Income: >23 k × 1 k–1.6 k	−1.0427	1.0786	−0.9668	0.3337
Overweight: yes × no	1.0418	0.4207	2.4765	0.0133

The statistical parametric model fitted to data to account for the probability of an impaired outcome in the MoCA-B test according to the diagnosis of COVID-19, participant age, income, and overweight status. The class of the model is specified in the table’s head. The estimated coefficients fitting an instance of such a model class are introduced in the “Estimate” column. Each coefficient represents the association between the corresponding predictor (as informed in the row’s label) and the result in the MoCA-B and is followed by the respective statistics from the Wald test for the evaluation of its statistical significance.

**Figure 2:**
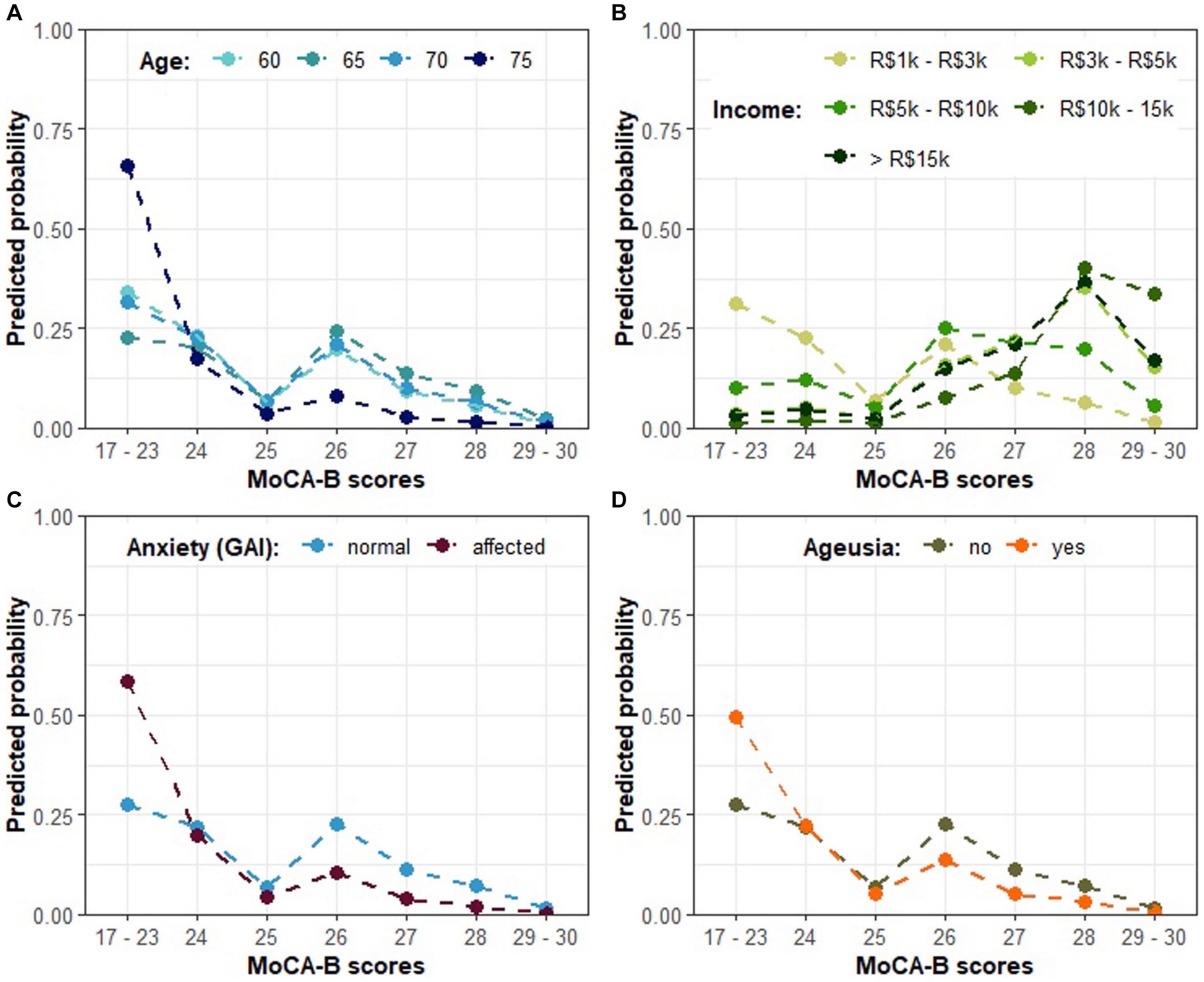
The predicted probability of each MoCA-B outcome on COVID-19-affected patients according to model A in Table 4. **(A)** The predicted probability of MoCA-B outcome according to COVID-19-affected patients’ age; **(B)** MoCA-B according to patients’ income; **(C)** MoCA-B according to anxiety; **(D)** MoCA-B according to the presence of ageusia. Individual plots illustrate the expected effect of changing levels of a single predictor, while the others are kept in the basal category reference (i.e., 67 years as the average for age, R$1k to R$3k for income, no anxiety according to GAI and absence of ageusia).

Once the association between COVID-19 and cognitive impairment as assessed by MoCA-B had been confirmed, we continued to investigate for further characteristics that could explain or predict the severity of the cognitive decline in the cohort of COVID-19-affected individuals. In this context, both age (Table 4) (Wald test: *p* < 0.001 and *p* = 0.0155 for the first and second order orthogonal polynomials for age, respectively) and income (Table 4) (Wald test: all *p* < 0.083) were ratified as statistically significant variables influencing MoCA-B score. While controlling for these variables, an association between anxiety as assessed by GAI and the performance on MoCA-B emerged (Table 4) (Wald test: estimate = −1.303, SE = 0.662, *Z* = −1.966, *p* = 0.049). In this regard, the odds for a greater MoCA-B score were estimated to decrease by 72.83% (OR = 0.271) among anxious individuals. In addition, while keeping all other factors fixed, the occurrence of ageusia during acute COVID-19 was also found to be a relevant predictor of the MoCA-B outcome, although the evidence supporting such an association remained marginally significant (Table 4) (Wald test: estimate = −0.939, SE = 0.489, *Z* = −1.920, *p* = 0.054). The presence of ageusia during COVID-19 was estimated to reduce the odds of a greater MoCA-B score by 60.93% (OR = 0.390). Figure 2A illustrates the influence of COVID-19 patients’ age on MoCA-B score, evidencing that individuals as older as 75 tend to prevail in the range of 17–23 (65.90% estimated probability), against a lower probability (around 30%) among patients between 60 and 70. Such influence was represented while income was fixed at the lowest reference level. When income increases, higher MOCA-B scores are expected (Figure 2B). It is likely that participants who experience anxiety score in the lowest range of MoCA- B (17–23; Figure 2C), so are participants who present with ageusia during acute COVID-19, although not so prominent (Figure 2D). At last, it is important to note that the predictions above are based on scenarios where only one predictor changes, while all others remain fixed at the basal reference level (i.e., 67 years for age, R$1 k to R$3 k for income, no anxiety according to GAI and absence of ageusia).

**Table 4:** CLM with logit link for MoCA-B (Wald test)—models 1 and 2.

	Estimate	Std. Error	*Z*	*p*
Model 1				
Age (linear)	−7.4552	2.1574	−3.4556	0.0005
Age (quadratic)	−5.3407	2.2074	−2.4195	0.0155
Income: 3 k–5 k × 1 k–3 k	2.4897	0.9101	2.7358	0.0062
Income: 5 k–10 k × 1 k–3 k	1.4012	0.8088	1.7325	0.0832
Income: 10 k-15 k × 1 k–3 k	3.5033	1.0045	3.4877	0.0005
Income: >15 k × 1 k–3 k	2.5973	0.9574	2.7129	0.0067
Anxiety (GAI): affected × normal	−1.3031	0.6626	−1.9665	0.0492
Covid symptom: ageusia	−0.9399	0.4893	−1.9208	0.0548
Model 2				
Habits: Cognitive stimulating courses	1.271	0.6828	1.8613	0.0627
Overweight	−2.2071	0.6029	−3.6607	0.0003
Covid symptom: ageusia	−1.0283	0.4789	−2.147	0.0318

The statistical parametric models fitted to data to account for the MoCA-B score according to the patient’s age, income, anxiety as assessed by GAI, occurrence of ageusia during COVID-19, patient’s habit of attending cognitive stimulating courses and overweight status. The class of the models are specified in the table’s head, along with the link function matching the expected cumulative probability of a given outcome to the linear combination of the predictors. The estimated coefficients fitting an instance of such a model class are introduced in the “Estimate” column. Each coefficient represents the association between the corresponding predictor (as informed in the row’s label) and the result in MoCA-B and is followed by the respective statistics from Wald test for the evaluation of its statistical significance. To avoid multicollinearity, the association with age was decomposed into linear and quadratic terms of an orthogonal polynomial.

**Figure 3:**
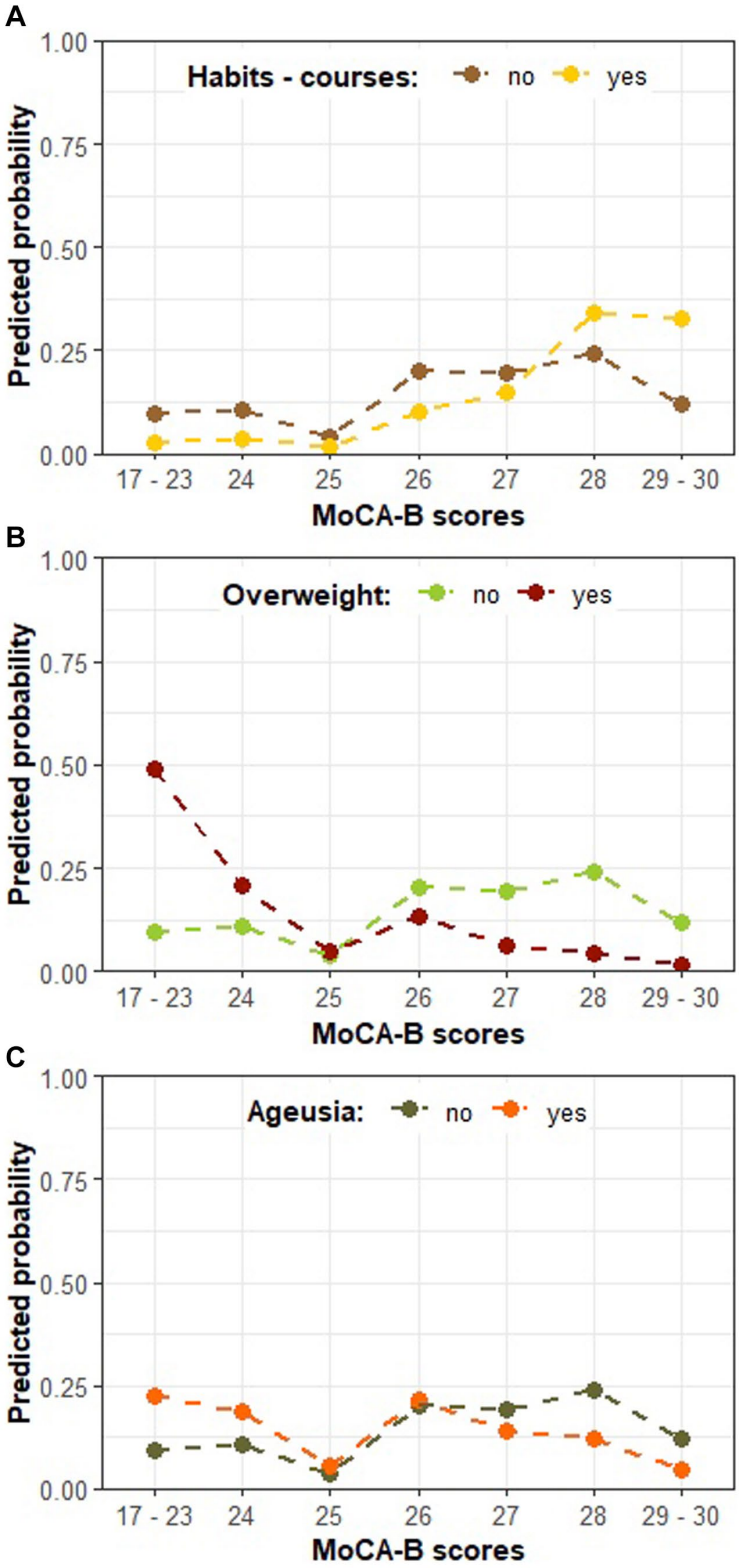
The predicted probability of each MoCA-B outcome on COVID-19-affected patients according to model B in Table 4. **(A)** The predicted probability of MoCA-B outcome according to the habit of cognitively stimulating courses; **(B)** MoCA-B according to patients’ weight classification; **(C)** MoCA-B according to the presence of ageusia; Individual plots illustrate the expected effect of changing levels of a single predictor, while the others are kept in the basal category reference (i.e., no engagement in cognitive stimulating courses, no overweight, and absence of ageusia).

In order to further explore individual characteristics influencing the MoCA-B performance, a second model described in Table 4 ratifies the association between ageusia and MoCA-B impairment (Wald test: estimate = −1.028, SE = 0.478, Z = −2.147, *p* = 0.031) and introduces new associations. In addition, the odds for a higher MoCA-B score were also estimated to reduce by 89.00% when a patient is overweight (Table 4; OR = −2.207, Wald test: estimate = −2.207, SE = 0.602, *Z* = −3.660, *p* < 0.001). Next, we found substantial evidence supporting an association between the habit of attending cognitively stimulating courses (cognitive training, foreign language or technology classes) and MoCA-B, although insufficient to overcome significance and remaining as a statistical trend (Table 4; Wald test: estimate = 1.2710, SE = 0.6828, *Z* = 1.8613, *p* = 0.0627). Nonetheless, the odds for a higher MoCA-B score were estimated to increase by 256.43% (OR = 1.2710) when a patient is regularly engaged in such courses compared to those seldom enrolled in equivalent brain stimulation activities. Cognitive stimulation was consistently indicated as a significant predictor along the modeling process, and it rather represents a set of stimulatory routines, such as the practice of traveling, for which statistical significance was also repeatedly confirmed. Some post-COVID symptoms, such as alopecia and arthralgia, were also indicated as embodying significant associations with the MoCA-B outcome. Nevertheless, such relations could not be explored in the present study, given the inherent space limitation for further complementary statistical models. Figure 3A describes the association between routine cognitive training programs and the MoCA-B performance, manifested as a strong inclination (65% probability) toward the highest scores (from 28 up to 30). In turn, such a profile is expected to shift markedly when COVID-19 patients are overweight, resulting in the virtual abolition of scores higher than 26 and a sharp increase in the probability of the lowest range of scores (Figure 3B). At last, ageusia was predicted to increase the probability of lower MoCA-B scores and reduce the probability of higher scores, whereas intermediate scores were likely to remain insensitive to this symptom.

## 4 Discussion

Our data points to cognitive sequelae in older adults 6 months after COVID-19 infection, as assessed by the MoCA-B test. After adjusting for multiple variables, the risk for an impaired MoCA-B was found to rely on the diagnosis of COVID-19, as well as on age, income, and body weight.

Individuals with previous COVID-19 infection had almost a 150% increase in the probability of cognitive impairment, even controlling for his/her age, income, and overweight status. This is consistent with the emerging literature on the subject. Del Bruto et al. compared MoCA results from an Ecuadorian cohort (mean age of 62.6 years) before and during the pandemic and demonstrated that individuals with serological evidence of previous exposure to SARS-CoV-2 increased the chance of cognitive decline by 18.1 times after adjusting for education, sleep quality, depression, and cardiovascular risk factors (17). The metanalysis conducted by Crivelli et al. compared MoCA results of COVID survivors and healthy controls and concluded that patients who recovered from COVID-19 experienced a decline in general cognition (≈1-point difference on MoCA) when compared to healthy controls, up to 7 months after infection (18). Nevertheless, controversies still exist regarding the link between COVID-19 and cognitive impairment; for instance, the study of Whiteside et al. did not show significant cognitive deficits in a battery of cognitive tests 6 months post-infection (19).

The precise mechanisms through which SARS-CoV-2 may impact cognition have yet to be synthesized. Cognitive function depends on precise neural circuit activity regulated by neurons and glial cells (4), and it is acknowledged that microglial and astrocyte reactivity plays a significant role in SARS-CoV-2-induced neuroinflammation. Therefore, growing evidence has been built in disentangling the association between neuroinflammation and imbalanced cerebral homeostasis, neurogenesis, and plasticity (20–22). Such events might trigger new or exacerbate preexisting neurodegenerative processes (23). Additionally, functional and structural neuroimaging studies suggest a variety of brain areas susceptible to SARS-CoV-2 neurotropism that have been linked to impaired cognitive performance (24), such as the prefrontal and frontoparietal (25), cerebellum (26), limbic and paralimbic regions (22, 25–27), the later possibly indicating a CNS SARS-CoV-2 route through olfactory pathways. Reinforcing this hypothesis, a number of studies have associated COVID-19 olfactory dysfunction with impaired cognitive measures and mood disturbances (26–28), suggesting similar mechanisms underpinning both conditions.

Interestingly, our results persistently evidenced that acute ageusia, not anosmia, predicted the severity of cognitive impairment in those previously in contact with SARS-CoV-2. Some of the explanations for acute ageusia include the viral activity on ACE2 receptors followed by inflammatory reactions in sialic receptors and taste buds, with reduced salivary output, edema, hypoxia, and apoptosis of taste buds, leading to atypical taste bud turnover and chemosensory impairment (29). In a Brazilian study of 701 hospitalized adults with moderate to severe COVID-19, those who experienced hypogeusia alone or concomitant with hyposmia performed worse on memory tests. The authors suggest that the division between taste and smell resides more on theory than practical issues, and a provisional explanation for the findings might again involve the interaction of SARS-CoV-2 with several brain structures linked to the olfactory cortices (30). In fact, gustatory and olfactory areas in the brain are often interrelated, and most apparent gustatory dysfunctions are the result of impaired olfaction rather than gustation (31).

Some sociodemographics implicated poorer performance on MoCA-B in our total sample and were also individual predictors of the severity of cognitive decline among those previously affected by COVID-19. It is known that aging is a major risk factor for cognitive impairment (32). We found a 15% increase in the odds of MoCA-B impairment for every age unit increment, in line with some previous COVID-19 research that indicates older age is associated with greater changes in cognition (33, 34). Lower income was strongly correlated with the risk for cognitive decline. Income is often considered an indicator of wealth and is generally associated with one’s level of education, occupational status, and access to health services (35). These factors often interrelate with lifestyle, cardiovascular disease, and cognitive reserve, all potentially modifiable risk factors for dementia (36). Cognitive reserve results from education, intellectually stimulating occupation, and various other activities across the lifespan and is thought to protect individuals against clinically significant cognitive decline even in the presence of neuropathology (37, 38). Cognitive training, a late-life intellectual stimulation, usually focuses on enhancing fluid cognitive abilities and is believed to add up to cognitive reserve through mechanisms that theoretically may expand cognitive capacity, increase cognitive efficiency, or both (39). In this perspective, COVID-19-affected patients who regularly engaged in cognitively stimulating activities—cognitive training, foreign language classes, or tech courses—performed better on MoCA-B. The evidence of the preventive role of cognitive training in dementia is controversial. A Chinese study followed up on more than 15,000 older adults for 5 years and found a 30% reduced risk of incident dementia in those participating in daily intellectual activities (40). Contrarily, some meta-analyses and systematic reviews found immediate improved cognition but no long-lasting effects, possibly due to heterogeneous interventions and outcome measures (41, 42).

Being overweight – one of the modifiable risk factors for dementia—had a significant negative impact on cognition in our sample (36). A systematic review of more than 500,000 adults followed up on obese adults for four decades and showed a 1.3 times greater risk of developing dementia (43). Interestingly, another meta-analysis found that a loss of 2 kg or more was associated with significant improvements in tests of attention and memory in obese adults over 50 years (44). Several pathways may account for the association between overweight/obesity and cognitive decline: adiposity accumulation results in the release of adipokines and insulin resistance, implicated in the genesis of diabetes, arterial hypertension, dyslipidemia, and cardiovascular diseases. These, in turn, are involved in changes in the brain structure and circulation, contributing to the installation of both the pathologies of AD and vascular dementia (45).

In the same direction as Delgado-Alonso et al. (46), having a diagnosis of anxiety but not depression increased the odds of poorer cognition among COVID-19 patients. A 2016 systematic review of longitudinal studies on anxiety disorders found a 6.5% increased risk of cognitive impairment in the community and a 7.9% increase in dementia risk, especially in the population aged 80 and over (47). According to the authors, anxiety can either be a prodrome of neurocognitive disorders or be a symptom in the course of neurodegeneration through a variety of mechanisms: (1) the state of hypercortisolism and consequent glucocorticoid hyperstimulation in medial temporal lobe receptors can result in hippocampal atrophy, increased amyloid production, and tau accumulation; (2) increased inflammatory cytokines in anxiety, for example, IL-6 and TNF, contributes to neuroinflammation; (3) patients with anxiety present lower production of neurotrophic factors like the Brain Derived Neurotrophic Factor (BDNF); (4) physiological reactions triggered by anxiety, such as increased blood pressure and heart rate, vasoconstriction and platelet hyperreactivity, culminates in cardiovascular diseases, known to be associated with cognitive disorders, and (5) lower cognitive reserve resulting from poorer mental and social stimuli usually experienced throughout life by those with anxiety disorders.

We found a higher prevalence of subjective cognitive complaints among COVID-19 participants compared to previous meta-analyses (57%) (48, 49), possibly justified by the older population in our study. Although there is increasing evidence supporting concerns about older adults’ subjective cognitive complaints (50), such complaints were not associated with poorer results in the objective cognitive screening. In the context of COVID-19, it seems that psychosocial burden and other social determinants of health may influence the perception of cognitive deficits even in the absence of objective dysfunction, as evidenced in previous studies (19, 51–53).

Currently, the MoCA test is widely used for screening cognitive decline, including in the context of COVID-19 (54, 55). We chose to adopt the `Basic’ version as this is better suited for individuals with lower educational levels, which is particularly common among Brazilian elders. Surprisingly, most of our sample - in both groups - had higher education and income levels, resulting in statistically disproportional subgroups concerning these sociodemographic variables. Nevertheless, both COVID and control participants scored near the threshold of cognitive decline in MoCA-B, despite their higher educational background; 35.7% of COVID-19 survivors against 20.3% of the control group were classified as cognitively impaired (*p* = 0.014). These numbers exceed the pre-COVID-19 expected prevalence of mild cognitive impairment (8.4%) (56) and dementia (1.2%) (57) for people aged 65–69 (mean age in our study). It could be hypothesized that the social isolation imposed by the COVID-19 pandemic triggered even higher levels of stress in the older population and greater vulnerability to illness and death, which possibly explains the increased proportion of individuals with low cognitive scores, even if not previously exposed to SARS-CoV-2 infection (58). Recently published systematic review and meta-analysis blamed prolonged social isolation as responsible for incident cognitive decline (59) and up to 60% greater risk of developing dementia in older adults (60).

One of the key strengths of this study is its focus on the older age group. Soon, the longitudinal nature of our data will help add crucial information about the long-term cognitive effect of SARS-CoV-2 infection on this population. Having a control group without previous serological SARS-CoV-2 exposure adds reliability to our work, as well as recruiting volunteers and collecting data before COVID-19 vaccination. Additionally, researchers were blind to the previous exposure of participants to SARS-CoV-2, which helped to reduce bias. Finally, our sample’s level of income and education may indicate that our results can be generalizable also to high-income countries (HIC). Hopefully, our findings may add important contributions to public health policies, targeting modifiable risk factors in the aging population toward cognitive decline risk reduction after COVID-19 infection. Nevertheless, we acknowledge some limitations in this study. First, it is important to note that the findings may not apply to younger individuals, as the study focused specifically on those 60 and older. Second, we recognize some possible selection biases, such as individuals with high income and educational levels and those with cognitive complaints; all these might have influenced greater interest in research volunteering. Third, the clinical data regarding comorbidities, medications, and COVID-19 infection was provided by the participants themselves instead of being obtained from medical records. Fourth, it should be noted that while both TICS-m and MoCA-B tests have been translated and adapted to Portuguese, there are currently no validated cutoffs for cognitive decline non-dementia in the general Brazilian population. It is worth noting that MoCA can be used as an initial cognitive screening tool, and a more comprehensive neuropsychological evaluation is imperative to understand the cognitive sequelae of COVID-19 infection fully.

## 5 Conclusion

Our study highlights a profile of cognitive risk aggravation over aging after COVID-19 infection, which is likely mitigated by wealth but worsened in the presence of overweight. At the same time, it suggests the importance of initiating interventions toward risk reduction, such as late-life engagement in cognitively stimulating activities, weight reduction, and treatment of psychiatric conditions. Further research is necessary to understand the nature of this condition fully and to determine whether specific cognitive functions might be affected on a long-term basis. It is imperative to prioritize studies on COVID-19 cognitive impairment in older adults, given their heightened vulnerability to cognitive disorders.

## Data availability statement

The raw data supporting the conclusions of this article will be made available by the authors, without undue reservation.

## Ethics statement

The studies involving humans were approved by Comitê de Ética em Pesquisa (CEP)—Campus Central—UFRN. The studies were conducted in accordance with the local legislation and institutional requirements. The participants provided their written informed consent to participate in this study.

## Author contributions

VP: Conceptualization, Data curation, Formal analysis, Investigation, Methodology, Project administration, Supervision, Validation, Visualization, Writing – original draft, Writing – review & editing. LF: Investigation, Methodology, Writing – original draft. TB: Investigation, Methodology, Writing – original draft. RS: Investigation, Methodology, Writing – original draft. AD: Investigation, Methodology, Writing – original draft. MS: Investigation, Data curation, Writing – review & editing. KA: Conceptualization, Funding acquisition, Methodology, Resources, Supervision, Writing – review & editing.
