# Supplementary material for: Factors associated with older adults’ cognitive decline 6 months after gamma-variant SARS-CoV-2 infection
Source: Front Neurol. 2024 Feb 15;15:1334161. doi: 10.3389/fneur.2024.1334161 (PMC10902427; doi:10.3389/fneur.2024.1334161)
Supplement: Supplementary file 1 [file Table_1.docx]

**Supplementary Table 1** – Characteristics of acute COVID-19 disease and persistent symptoms 6 months after diagnosis in the COVID group

| Clinical characteristics of COVID-19 | COVID group  (n = 70) | |
| --- | --- | --- |
| **Acute disease - CNS related symptoms – n (%)**  Ageusia  Anosmia  Headache  Confusion | | 24 (34.3)  22 (31.4)  22 (31.4)  06 (8.6) |
| **Acute disease – Complications – n (%)**  Pneumonia  In-hospital treatment  Supplementary O2 | | 38 (54.3)  16 (22.9)  16 (22.9) |
| **Long COVID symptoms – n (%)**  Cognitive complaints  Alopecia  Arthralgia  Fatigue  Myalgia  Insomnia  Hearing problems  Asthenia  New onset-mood symptoms | | 35 (50.0)  11 (15.7)  08 (11.4)  05 (7.1)  03 (4.3)  02 (2.8)  02 (2.8)  02 (2.8)  02 (2.8) |

Values expressed as number of participants (n)/frequencies (%).
