# Supplementary material for: Factors associated with older adults’ cognitive decline 6 months after gamma-variant SARS-CoV-2 infection
Source: Front Neurol. 2024 Feb 15;15:1334161. doi: 10.3389/fneur.2024.1334161 (PMC10902427; doi:10.3389/fneur.2024.1334161)
Supplement: Supplementary file 2 [file Table_2.docx]

**Supplementary Table 2** - Comparison of the prevalence of self-reported comorbidities and medications in use between the COVID (at the time of collection) and CONTROL groups.

|  | COVID  (n = 70)    n (%) | CONTROL  (n = 153)      n (%) | Statistics | *p* value |
| --- | --- | --- | --- | --- |
| **Comorbidities** |  |  |  |  |
| Dyslipidemia | 44 (62.9) | 82 (53.6) | 1.676^(2)^ | 0.195 |
| Hypertension | 35 (50.0) | 87 (56.9) | 0.913^(2)^ | 0.339 |
| Joint diseases | 25 (35.7) | 67 (43.8) | 1.293^(2)^ | 0.256 |
| Diabetes | 17 (24.3) | 33 (21.6) | 0.204^(2)^ | 0.652 |
| Overweight | 13 (18.6) | 26 (17.0) | 0.083^(2)^ | 0.773 |
| Anxiety | 13 (18.6) | 34 (22.2) | 0.385^(2)^ | 0.535 |
| Hypothyroidism | 13 (18.6) | 20 (13.1) | 1.152^(2)^ | 0.283 |
| Osteoporosis | 13 (18.6) | 35 (22.9) | 0.527^(2)^ | 0.468 |
| Hystory of cancer | 12 (17.1) | 20 (13.1) | 0.648^(2)^ | 0.421 |
| Glucose intolerance | 11 (15.7) | 29 (19.0) | 0.343^(2)^ | 0.558 |
| Arrhythmia | 10 (14.3) | 20 (13.1) | 0.061^(2)^ | 0.805 |
| Coronary disease | 09 (12.9) | 14 (9.2) | 0.713^(2)^ | 0.398 |
| Depression | 09 (12.9) | 18 (11.8) | 0.054^(2)^ | 0.816 |
| **Medications** |  |  |  |  |
| Statins | 42 (60.0) | 69 (45.1) | 4.266^(2)^ | **0.039** |
| ACEi/ARB | 27 (38.6) | 64 (41.8) | 0.211^(2)^ | 0.646 |
| Vitamin D | 22 (31.4) | 63 (41.2) | 1.935^(2)^ | 0.164 |
| Oral antidiabetics | 20 (28.6) | 52 (34.0) | 0.644^(2)^ | 0.422 |
| Antidepressant | 18 (25.7) | 29 (19.0) | 1.319^(2)^ | 0.251 |
| Betablockers | 18 (25.7) | 37 (24.2) | 0.061^(2)^ | 0.806 |
| Platelet antiaggregants | 15 (21.4) | 17 (11.1) | 4.160^(2)^ | **0.041** |
| Thyroid hormone | 14 (20.0) | 23 (15.0) | 0.856^(2)^ | 0.355 |
| Benzodiazepines | 09 (12.9) | 23 (15.0) | 0.185^(2)^ | 0.667 |
| Diuretics | 09 (12.9) | 27 (17.6) | 0.814^(2)^ | 0.367 |
| Proton pump inhibitors | 08 (11.4) | 16 (10.5) | 0.047^(2)^ | 0.828 |
| Psychoactive drugs | 21 (30.0) | 46 (30.0) | 0.000^(2)^ | 0.992 |

Values expressed as the number of individuals (n) / frequencies (%). Comorbidities listed with prevalence >5% in descending order of frequency according to COVID group. (2) Chi-square (X2) test for differences between groups. Significant results (p < 0.05) in bold. Abbreviations: COVID, individuals with previous COVID-19; ACEi, angiotensin-converting enzyme inhibitors; ARB, angiotensin II receptor blockers.
